# Mutations in epigenetic regulator *KMT2C* detected by liquid biopsy are associated with worse survival in prostate cancer patients

**DOI:** 10.32604/or.2023.028321

**Published:** 2023-06-27

**Authors:** SHA ZHU, NANWEI XU, JIAYU LIANG, FENGNIAN ZHAO, ZILIN WANG, YUCHAO NI, JINDONG DAI, JINGE ZHAO, XINGMING ZHANG, JUNRU CHEN, GUANGXI SUN, PENGFEI SHEN, HAO ZENG

**Affiliations:** Department of Urology, Institute of Urology, West China Hospital, Sichuan University, Chengdu, 610041, China

**Keywords:** KMT2C, Epigenetic regulator, Liquid biopsy, Prostate cancer, Survival, Biomarker

## Abstract

**Background:**

KMT2 (lysine methyltransferase) family enzymes are epigenetic regulators that activate gene transcription. *KMT2C* is mainly involved in enhancer-associated H3K4me1, and is also one of the top mutated genes in cancer (6.6% in pan-cancer). Currently, the clinical significance of *KMT2C* mutations in prostate cancer is understudied.

**Methods:**

We included 221 prostate cancer patients diagnosed between 2014 and 2021 in West China Hospital of Sichuan University with cell-free DNA-based liquid biopsy test results in this study. We investigated the association between *KMT2C* mutations, other mutations, and pathways. Furthermore, we evaluated the prognostic value of *KMT2C* mutations, measured by overall survival (OS) and castration resistance-free survival (CRFS). Also, we explored the prognostic value of *KMT2C* mutations in different patient subgroups. Lastly, we investigated the predictive value of *KMT2C* mutations in individuals receiving conventional combined anti-androgen blockade (CAB) and abiraterone (ABI) as measured by PSA progression-free survival (PSA-PFS).

**Results:**

The *KMT2C* mutation rate in this cohort is 7.24% (16/221). *KMT2C*-mutated patients showed worse survival than *KMT2C*-wild type (WT) patients regarding both CRFS and OS (CRFS: mutated: 9.9 *vs*. WT: 22.0 months, *p* = 0.015; OS: mutated: 71.9 *vs*. WT 137.4 months, *p* = 0.012). *KMT2C* mutations were also an independent risk factor in OS [hazard ratio: 3.815 (1.461, 9.96), *p* = 0.006] in multivariate analyses. Additionally, we explored the association of *KMT2C* mutations with other genes. This showed that *KMT2C* mutations were associated with Serine/Threonine-Protein Kinase 11 (*STK11*, *p* = 0.004) and Catenin Beta 1 (*CTNNB1*, *p* = 0.008) mutations. In the CAB treatment, *KMT2C*-mutated patients had a significantly shorter PSA-PFS compared to *KMT2C*-WT patients. (PSA-PFS: mutated: 9.9 *vs*. WT: 17.6 months, *p* = 0.014). Moreover, *KMT2C* mutations could effectively predict shorter PSA-PFS in 10 out of 23 subgroups and exhibited a strong trend in the remaining subgroups.

**Conclusions:**

*KMT2C*-mutated patients showed worse survival compared to *KMT2C*-WT patients in terms of both CRFS and OS, and *KMT2C* mutations were associated with *STK11* and *CTNNB1* mutations. Furthermore, *KMT2C* mutations indicated rapid progression during CAB therapy and could serve as a potential biomarker to predict therapeutic response in prostate cancer.

## Background

Prostate cancer is the most frequent non-skin malignancy in men [1]. Next-generation sequencing techniques facilitate the possibility of tailoring clinical treatment based on various molecular characteristics of patients, which is especially important in the case of prostate cancer since it is a highly heterogeneous disease. Therefore, deciphering the mutations associated with treatment responses or patient outcomes is essential in targeted therapy.

KMT2 (lysine methyltransferase) family enzymes regulate gene transcription via post-translational histone methylation. More specifically, KMT2 enzymes methylate histone 3 lysine 4 (H3K4) on promoters (di- and tri-methylation, me2/me3) or enhancers (mono-methylation, me1), leading to increased genome accessibility and thereby activating gene transcription [2]. The transcription activation function of the KMT2 family is essential in normal cell physiology and mammalian development [3]. However, cancer cells are known to hijack normal development-related machinery for their own malignant evolution and proliferation [4]. Frequent dysregulation of KMT2 family function in cancer is precisely an example of this malignant transformation [5]. The KMT2 family in *Homo sapiens* (originally named mixed-lineage leukemia (MLL) family) are a highly conserved group of proteins, including MLL1/KMT2A, MLL2/KMT2B, MLL3/KMT2C, MLL4/KMT2D, KMT2F (SET1A), and KMT2G (SET1B) [5]. *KMT2C/KMT2D* are mainly involved in enhancer-associated H3K4me1 [5], and they are also the most frequently mutated genes in cancer [6], especially *KMT2C*, with a mutation rate in pan-cancer being 6.6% [7].

Missense mutations are the most common *KMT2C* mutation type. They contribute to tumorigenesis through dysregulating genome enhancer activity, which could lead to a previously fine-tuned network regulated by oncoproteins and tumor suppressors being disrupted [5]. *KMT2C* mutations have been reported to correlate with patient prognosis in many cancer types. However, it is currently unclear whether KMT2C works as a tumor suppressor or as an oncogene. In most cancers, e.g., medulloblastoma [8], lung cancer [9], head and neck cancer [10], gastric cancer [11], squamous cell carcinoma [12], *KMT2C* mutations or low expression of *KMT2C* correlate with worse survival outcomes, whereas in tumors like breast cancer and pancreatic ductal adenocarcinoma, conflicting prognostic evidence exists [13–17].

Besides prognosis significance, mechanistically, KMT2C may promote the transcription of DNA damage response (DDR) pathway genes and PD-L1 [18,19]. Thus its loss of function may confer sensitivity to poly (ADP-ribose) polymerase inhibitors (PARPis) and immune checkpoint inhibitors (ICIs). There are also clinical observations about the predictive value of *KMT2C* mutations for PARPis and ICIs [9,18,20,21].

Meanwhile, although *KMT2C* dysregulation plays an oncogenic role and is also a top mutated gene in prostate cancer (7%) [22,23], the clinical significance of *KMT2C* mutations in prostate cancer is currently understudied. Generally, there have been few attempts to explore the clinical value of *KMT2C* mutations in prostate cancer cohorts. One recent study investigated the biological consequences of *KMT2C* alterations in prostate cancer cells. Here, they used data from the International Cancer Genome Consortium (ICGC) and found that *KMT2C* truncating mutations were associated with reduced disease-free survival in prostate cancer [24]. However, probably due to the heterogeneity of data sources and lack of complete follow-up information, the mutation rate of ICGC (1.5%) does not reflect the actual distribution of *KMT2C* mutations in prostate cancer [22]. Thus, this finding needs further validation.

Our institute has a large database of prostate cancer patients with liquid biopsy genomic test results. We keep records of their baseline information, disease progression, treatment outcomes, and genomic data. Thus, inspired by the research mentioned above, we analyzed the association between *KMT2C* mutation status and patients’ survival using data from our institute to further address the significance of *KMT2C* mutations in prostate cancer. Our results clearly suggest an adverse predictive and prognostic value of *KMT2C* mutations. Furthermore, we also discovered two gene mutations associated with *KMT2C* mutations. Lastly, *KMT2C* mutations were significantly associated with poor survival in patients with specific signaling pathway aberrations, suggesting further research should focus on the *KMT2C*-mediated epigenetic regulation of these pathways.

## Methods

### Patients

Prostate cancer patients diagnosed between 2014 and 2021 in West China Hospital of Sichuan University with liquid biopsy test results were included in this study. Other inclusion criteria are available from detailed pathological reports in our institute, having acceptable patient compliance and being willing to complete the follow-up regularly. Exclusion criteria are concurrent second or multiple tumors from other origins, pathological subtype as neuroendocrine prostate cancer, and being unable to give informed consent. A total of 221 patients fulfilled our requirements and were included in this study. All included patients’ demographic and clinicopathological data, including date of birth, baseline prostate-specific antigen (PSA), *de novo* metastatic status, pathological reports, etc., were collected from the electronic medical record system. Venous blood was taken from each patient at the time of disease diagnosis. All patients provided written informed consent to participate in this study, which was conducted following the Declaration of Helsinki. The protocols in this study had the approval of the West China Hospital institutional review board in December 2021 (version number: 2021-1703).

### cfDNA sequencing

Blood samples were collected in cell-free DNA tubes (BEAVER, 43803, Jiangsu, China). The plasma was harvested by centrifuging at 1600 *g* for 10 min, aliquoted in 1.5 mL microtubes, followed by another centrifugation for 10 min of 16000 *g*, and then stored at −80°C. We used QIAamp Circulating Nucleic Acid Kit (Qiagen, Hilden, Germany) to extract the cell-free DNA (cfDNA) from plasma. The buffy coat was used for genomic DNA (gDNA) extraction. QIAamp DNA Mini Kit (Qiagen) was used to extract DNA from the white blood cells.

Kapa Hyper Prep Kit (Kapa Biosystems, Wilmington, MA, USA) was applied to construct the sequencing library using 30–60 ng cfDNA, combined with Unique Molecular Identifier (UMI) to lower the false-positive rate of variant calls and increase variant detection sensitivity. Final libraries were sequenced on Illumina Nextseq500 (PE 75) (Illumina, San Diego, CA, USA) according to the manufacturer’s instructions.

For the liquid biopsy, cfDNA was estimated by a targeted sequencing strategy capturing all exons of 150 PCa-related genes (Suppl. Table 1). NGS was performed in 3Dmed Clinical Laboratory, a College of American Pathologists (CAP) and Clinical Laboratory Improvement Amendments (CLIA) certified laboratory of 3D Medicines Inc. All mutations were interpreted as pathogenic or likely pathogenic mutations according to American College of Medical Genetics and Genomics criteria. The supplementary table contains specific information about which signaling pathway the gene belongs to (Suppl. Table 1). Additionally, the details of the pathogenic (and probably pathogenic) variants detected in this study are provided in the Suppl. Table 2.

The paired-end reads were mapped by BWA-MEM algorithm [25]. A filtering model for the accuracy of mutation calling was applied as described before [26]. Single-nucleotide variations (SNVs), small insertion/deletions (indels) were called using MuTect [26], the union of Varscan 2 [27] and Pindel [28]. To increase the result accuracy, the mutations were further reviewed by Integrative Genomics Viewer (IGV) [29]. All mutations were manually reviewed using IGV to eliminate false-positive results further. We calculated mutant ctDNA and wild-type (WT) cfDNA fragments’ probability density distributions by Gaussian kernel smoothing using StatsModels [30], a Python library (v0.13.5 under python v3.7).

### Follow-up

Patients were required to visit our department every three weeks for routine history-taking, physical examinations, and PSA tests. Computerized tomography (CT), Magnetic resonance imaging (MRI), and single-photon emission computed tomography (SPECT) were performed when necessary.

### Statistics

This study used PSA progression-free survival (PSA-PFS), castration resistance-free survival (CRFS), and overall survival (OS) as primary endpoints. According to the PCWG3 criteria [31], PSA-progression was defined as two consecutive rises in the PSA level of 25% or more above the nadir (and by ≥2 ng/ml) after the treatment initiation. We used the chi-square and rank-sum tests to compare baseline characteristic differences between groups. Kaplan-Meier curve and log-rank test were used for survival curves. We used the Cox regression model for univariate and multivariate survival analyses. The impact of all variables on the outcome of the patients was determined through multivariate analyses using the “enter” method. All tests were two-sided. All statistical analyses were performed by R version 4.0.5 using the R package tableone v0.13.2, readxl v1.4.1, survival v3.4.0, plyr v1.8.6, forestplot v3.1.0, survminer v0.4.9, ggplot2 v3.3.5, gridExtra v2.3, patchwork v1.1.2. A *p* < 0.05 was considered significant.

## Results

### Study design and patient characteristics

We retrospectively enrolled 221 prostate cancer patients with liquid biopsy genomic test results in this study. Most patients belonged to high-risk or advanced groups: baseline PSA >100 ng/mL: 61.40% (132/221); *de novo* metastasis: 89.14% (197/221); International Society of Urological Pathology (ISUP) grade ≥4: 81.45% (180/221). All baseline characteristics were balanced between *KMT2C*-WT and *KMT2C*-mutated groups, including baseline PSA, age, metastasis at different sites, and ISUP grade (Table 1).

**Table 1:** Baseline characteristics of all patients, patients with or without *KMT2C* mutations

		Overall (n = 221)	*KMT2C*-WT (n = 205)	*KMT2C*-mutated (n = 16)	*p* value
Baseline PSA (median [IQR])		100.100 [48.000, 183.000]	100.100 [47.750, 183.000]	100.100 [84.787, 123.825]	0.9722
Baseline PSA (ng/mL, %)	**<100**	89 (40.27)	85 (41.46)	4 (25.00)	0.3037
	**≥100**	132 (59.73)	120 (58.54)	12 (75.00)	
Age (median [IQR])		69.000 [62.000, 75.000]	69.000 [63.000, 75.000]	65.500 [59.000, 72.000]	0.3639
Age (%)	**<70**	128 (57.92)	119 (58.05)	9 (56.25)	1
	**≥70**	93 (42.08)	86 (41.95)	7 (43.75)	
*De novo* metastasis (%)	**No**	24 (10.86)	22 (10.73)	2 (12.50)	1
	**Yes**	197 (89.14)	183 (89.27)	14 (87.50)	
PSA response to CAB (%)	**<50%**	28 (12.67)	28 (13.66)	0 (0.00)	0.2334
	**≥50%**	193 (87.33)	177 (86.34)	16 (100.00)	
ISUP grade (%)	**<4**	41 (18.55)	40 (19.51)	1 (6.25)	0.1959
	**≥4**	180 (81.45)	165 (80.49)	15 (93.75)	
AR pathway mutations (%)	**No**	167 (75.57)	158 (77.07)	9 (56.25)	0.1176
	**Yes**	54 (24.43)	47 (22.93)	7 (43.75)	
Cell cycle pathway mutations (%)	**No**	157 (71.04)	147 (71.71)	10 (62.50)	0.62
	**Yes**	64 (28.96)	58 (28.29)	6 (37.50)	
DDR pathway mutations (%)	**No**	169 (76.47)	157 (76.59)	12 (75.00)	1
	**Yes**	52 (23.53)	48 (23.41)	4 (25.00)	
MAPK pathway mutations (%)	**No**	195 (88.24)	182 (88.78)	13 (81.25)	0.6188
	**Yes**	26 (11.76)	23 (11.22)	3 (18.75)	
NED pathway mutations (%)	**No**	209 (94.57)	193 (94.15)	16 (100.00)	0.6727
	**Yes**	12 (5.43)	12 (5.85)	0 (0.00)	
PI3K pathway mutations (%)	**No**	186 (84.16)	174 (84.88)	12 (75.00)	0.4922
	**Yes**	35 (15.84)	31 (15.12)	4 (25.00)	
WNT pathway mutations (%)	**No**	196 (88.69)	184 (89.76)	12 (75.00)	0.1661
	**Yes**	25 (11.31)	21 (10.24)	4 (25.00)	
Total mutations (%)	**No**	94 (42.53)	94 (45.85)	0 (0.00)	0.0009
	**Yes**	127 (57.47)	111 (54.15)	16 (100.00)	

Note: PSA: prostate-specific antigen; CAB: combined anti-androgen blockade; ISUP: International Society of Urological Pathology; AR: androgen receptor; DDR: DNA damage response; MAPK: mitogen-activated protein kinase; NED: neuroendocrine differentiation; PI3K: phosphatidylinositol 3-kinase; IQR: interquartile range; WT: wild type.

In the hormone-sensitive prostate cancer (HSPC) stage, 185 patients were treated with conventional combined anti-androgen blockade (CAB). At the end of the follow-up, 164 patients had progressed to castration-resistant prostate cancer (CRPC), 137 of whom had received ABI treatment. Baseline characteristics were balanced and comparable between *KMT2C*-WT and *KMT2C*-mutated groups in patients who were still in the HSPC stage as well as those who had advanced to the CRPC stage (Suppl. Table 3).

### KMT2C mutations

The *KMT2C* mutation rate in this cohort is 7.24% (16/221), consistent with a previous report with an extensive database of 1,013 prostate cancer exome sequencing data [22]. Also, mutations in other pathways, including androgen receptor (AR) signaling pathway, cell cycle pathway, DDR pathway, mitogen-activated protein kinase (MAPK) pathway, neuroendocrine differentiation (NED) related pathway, phosphatidylinositol 3-kinase (PI3K) pathway, WNT pathway, did not differ between these two groups (Table 1).

However, a correlation between *KMT2C* mutations and Serine/Threonine-Protein Kinase 11 (*STK11*, *p* = 0.004) mutations and Catenin Beta 1 (*CTNNB1*, *p* = 0.008) mutations was found (Suppl. Table 4). Interestingly, when we split the entire cohort into two groups based on the patient’s current stage, we found that *KMT2C* mutations were associated with *STK11* (*p* = 0.043) mutation in the patients who had not progressed. Moreover, *KMT2C* mutations were correlated with *CTNNB1* (*p* = 0.012) and peckle-type poxvirus and zinc-finger protein (*SPOP*, *p* = 0.007) mutations in those that had advanced to CRPC (Suppl. Table 4).

### Survival analyses

**Figure 1:**
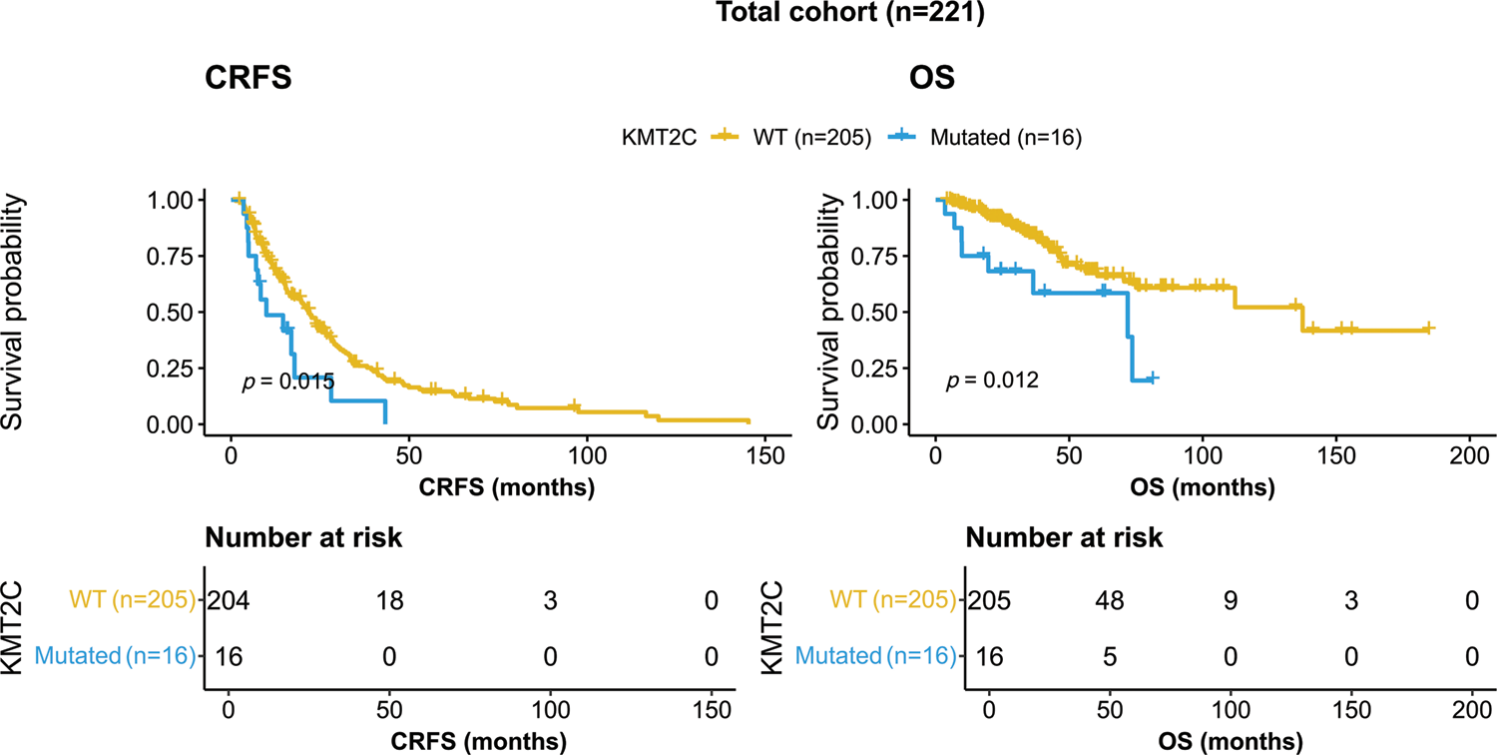
Kaplan-Meier curves of patients with or without *KMT2C* mutations. CRFS: castration-resistance free survival; OS: overall survival; WT: wild type.

*KMT2C*-mutated patients showed worse survival than *KMT2C*-WT patients in terms of both CRFS and OS (CRFS: mutated: 9.9 *vs*. WT: 22.0 months, *p* = 0.015; OS: mutated: 71.9 *vs*. WT: 137.4 months, *p* = 0.012, Fig. 1).

Furthermore, we did univariate and multivariate Cox regression analyses to explore the prognostic significance of *KMT2C* mutations, mutations in other pathways, and other clinicopathological indexes. The results showed that *KMT2C* mutations were associated with shorter CRFS and OS in univariate analyses, and were also an independent risk factor in OS [hazard ratio (HR): 3.815 (95% confidence interval (95% CI): 1.461, 9.96), *p* = 0.006], along with mutations in cell cycle pathway [HR: 2.231 (95% CI: 1.021, 4.876), *p* = 0.044] and neuroendocrine differentiation [HR: 3.633 (95% CI: 1.013, 13.027), *p* = 0.048] (Table 2). Although multiple factors were associated with shorter CRFS in univariate analyses, such as *KMT2C* mutations (*p* = 0.019), *de novo* metastasis (*p* = 0.020), AR pathway mutations (*p* = 0.002), MAPK pathway mutations (*p* = 0.001), PI3K pathway mutations (*p* = 0.008), WNT pathway mutations (*p* = 0.006), and total mutations (*p* = 0.015), none of them remained the prognostic significance in multivariate analyses for CRFS (only *KMT2C* mutations and *de novo* metastasis displayed borderline significance; *p* = 0.059 and *p* = 0.067).

**Table 2:** Univariate and multivariate survival analyses

	OS						CRFS					
	Univariate			Multivariate			Univariate			Multivariate		
	HR	95.0% CI	*p*	HR	95.0% CI	*p*	HR	95.0% CI	*p*	HR	95.0% CI	*p*
*KMT2C* mutated	2.56	(1.2–5.48)	0.015	3.82	(1.461, 9.96)	0.006	1.98	(1.12–3.51)	0.019	1.86	(0.976, 3.561)	0.059
PSA ≥100 ng/mL	1.2	(0.68–2.11)	0.532	0.96	(0.502, 1.845)	0.908	1.1	(0.8–1.5)	0.563	1.05	(0.736, 1.484)	0.807
Age ≥70	0.87	(0.5–1.53)	0.629	0.78	(0.426, 1.433)	0.426	0.88	(0.64–1.2)	0.412	0.88	(0.632, 1.226)	0.451
*De novo* metastasis	2.61	(1.01–6.73)	0.047	2.31	(0.847, 6.285)	0.102	1.76	(1.1–2.84)	0.02	1.62	(0.966, 2.725)	0.067
ISUP ≥4	2.57	(1.01–6.52)	0.047	1.69	(0.631, 4.536)	0.296	1.43	(0.95–2.17)	0.088	1.29	(0.837, 1.974)	0.252
PSA response	0.56	(0.28–1.12)	0.101	0.66	(0.275, 1.581)	0.351	0.73	(0.47–1.13)	0.16	0.71	(0.43, 1.174)	0.182
AR pathway	2.98	(1.71–5.19)	0	1.66	(0.719, 3.85)	0.234	1.7	(1.21–2.38)	0.002	1.00	(0.659, 1.53)	0.983
Cell cycle pathway	2.87	(1.65–4.99)	0	2.23	(1.021, 4.876)	0.044	1.28	(0.91–1.79)	0.15	0.96	(0.638, 1.429)	0.822
DDR pathway	1.69	(0.92–3.12)	0.09	1.18	(0.557, 2.497)	0.666	1.31	(0.92–1.87)	0.136	1.00	(0.657, 1.53)	0.991
MAPK pathway	3.6	(1.81–7.15)	0	1.67	(0.773, 3.596)	0.192	2.21	(1.38–3.54)	0.001	1.40	(0.888, 2.195)	0.148
NED pathway	2.44	(0.97–6.16)	0.059	3.63	(1.013, 13.027)	0.048	1.59	(0.86–2.93)	0.14	1.00	(0.418, 2.386)	0.998
PI3K pathway	2.61	(1.44–4.71)	0.002	2.04	(0.988, 4.218)	0.054	1.7	(1.15–2.52)	0.008	1.12	(0.771, 1.635)	0.546
WNT pathway	1.28	(0.54–3.02)	0.569	0.47	(0.141, 1.565)	0.219	1.86	(1.19–2.89)	0.006	0.66	(0.346, 1.255)	0.204
Total mutations	3.95	(1.97–7.91)	0	0.82	(0.481, 1.394)	0.461	1.48	(1.08–2.03)	0.015	1.13	(0.918, 1.382)	0.253

Note: OS: overall survival; CRFS: castration-resistance free survival; PSA: prostate-specific antigen; ISUP: International Society of Urological Pathology; AR: androgen receptor; DDR: DNA damage response; MAPK: mitogen-activated protein kinase; NED: neuroendocrine differentiation; PI3K: phosphatidylinositol 3-kinase; HR: hazard ratio; CI: confidence interval.

### Subgroup analyses

**Figure 2:**
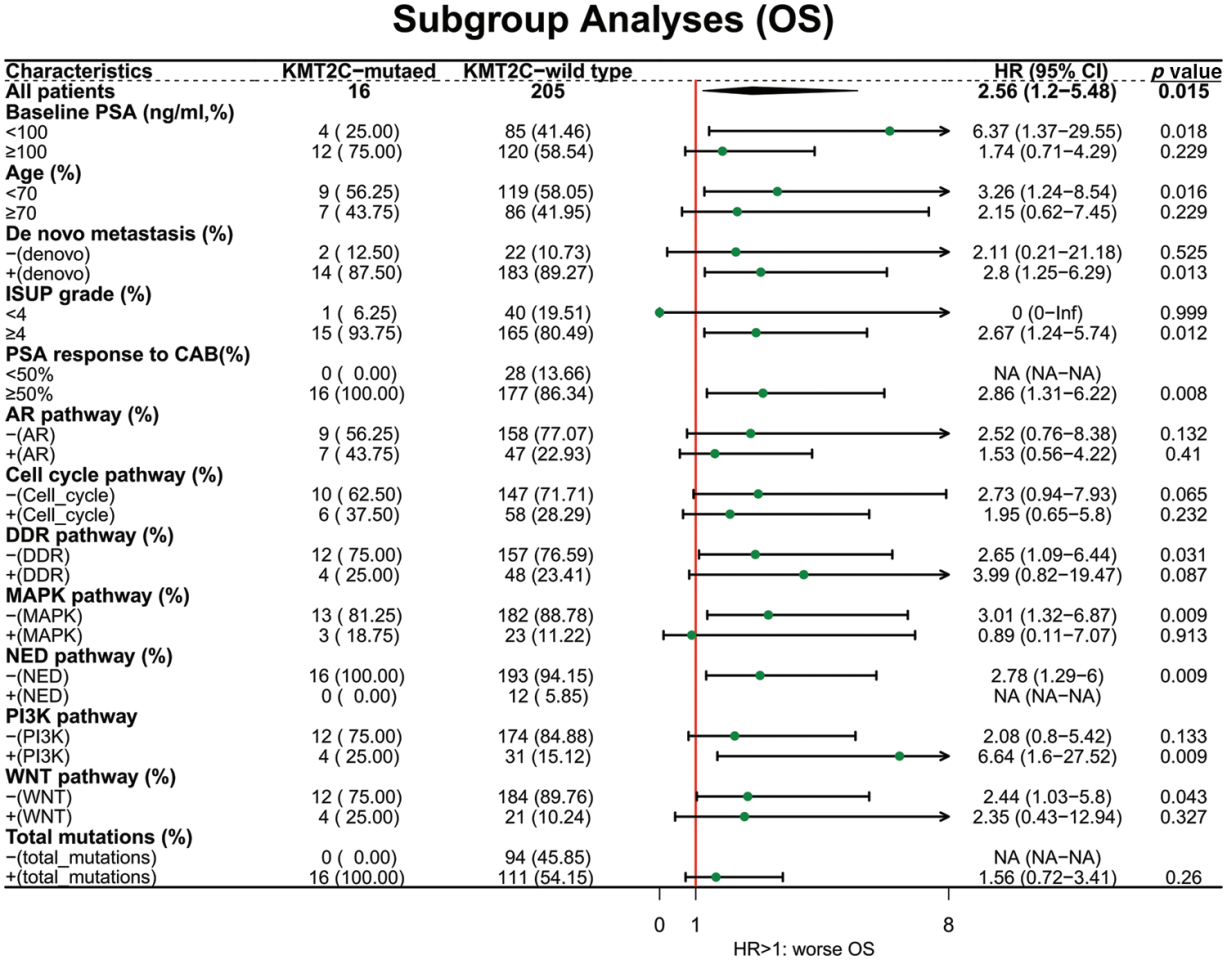
The prognostic value of *KMT2C* mutations in different clinicopathological subgroups. OS: overall survival; PSA: prostate-specific antigen; CAB: combined anti-androgen blockade; ISUP: International Society of Urological Pathology; AR: androgen receptor; DDR: DNA damage response; MAPK: mitogen-activated protein kinase; NED: neuroendocrine differentiation; PI3K: phosphatidylinositol 3-kinase; HR: hazard ratio; CI: confidence interval.

Next, we explored the prognostic value of *KMT2C* mutations in different patient subgroups. For OS, *KMT2C* mutations were associated with survival in patient subgroups with baseline PSA <100 ng/mL (HR: 6.37, *p* = 0.018), age <70 (HR: 3.26, *p* = 0.016), *de novo* metastasis (HR: 2.80, *p* = 0.013), ISUP grade ≥4 (HR: 2.67, *p* = 0.012), PSA response to CAB ≥50% (HR: 2.86, *p* = 0.008), no DDR pathway mutation (HR: 2.65, *p* = 0.031), without MAPK pathway mutation (HR: 3.01, *p* = 0.009), without NED pathway mutation (HR: 2.78, *p* = 0.009), with PI3K pathway mutation (HR: 6.64, *p* = 0.009), with WNT pathway mutation (HR: 2.44, *p* = 0.043) (Fig. 2).

### KMT2C mutations and therapeutic response

**Figure 3:**
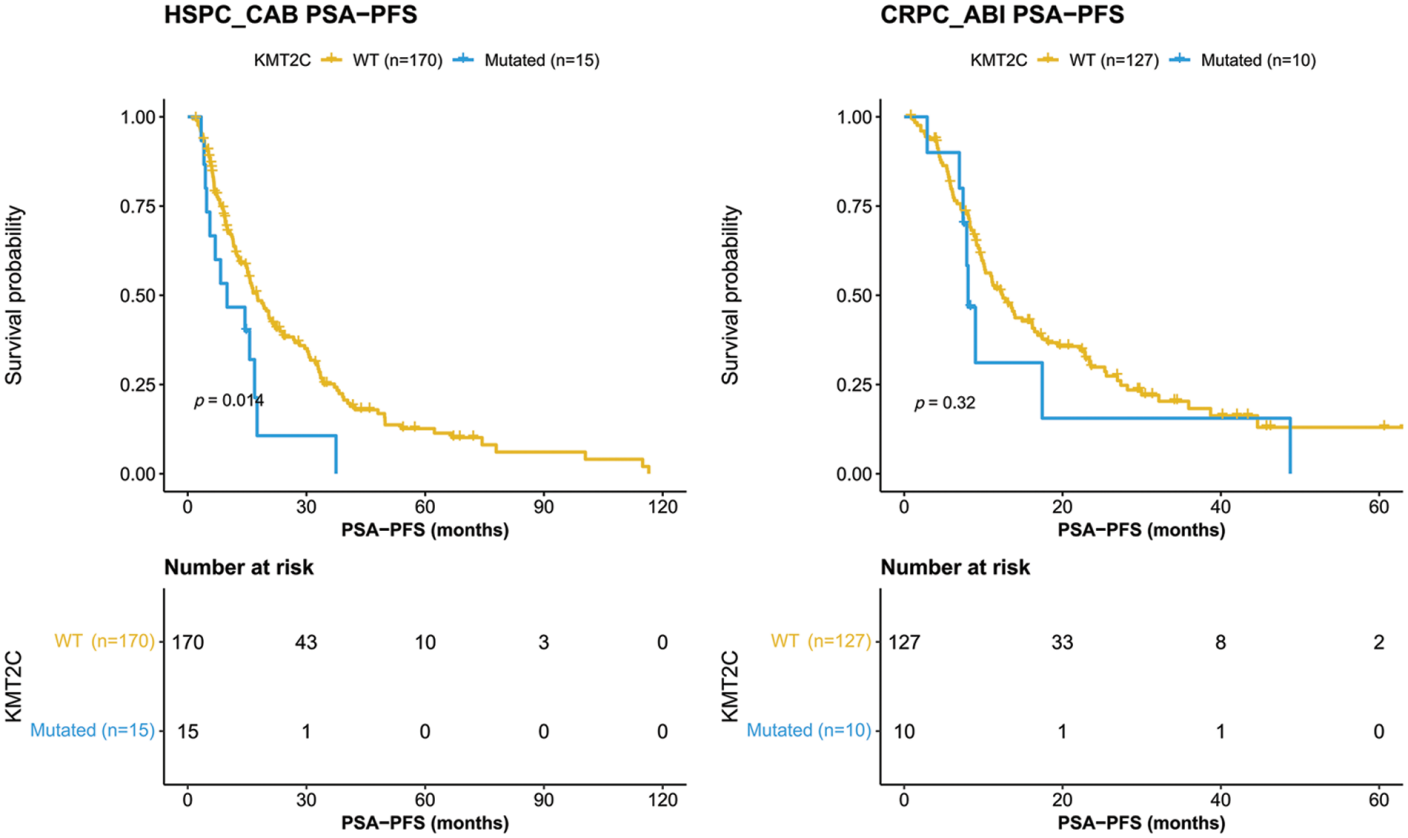
Kaplan-Meier curves of patients with or without *KMT2C* mutations who were treated combined anti-androgen blockade or abiraterone treatment. HSPC: hormone-sensitive prostate cancer; CRPC: castration-resistant prostate cancer; CAB: combined antiandrogen blockade; ABI: abiraterone; PSA: prostate-specific antigen; PSA-PFS: PSA progression-free survival.

**Figure 4:**
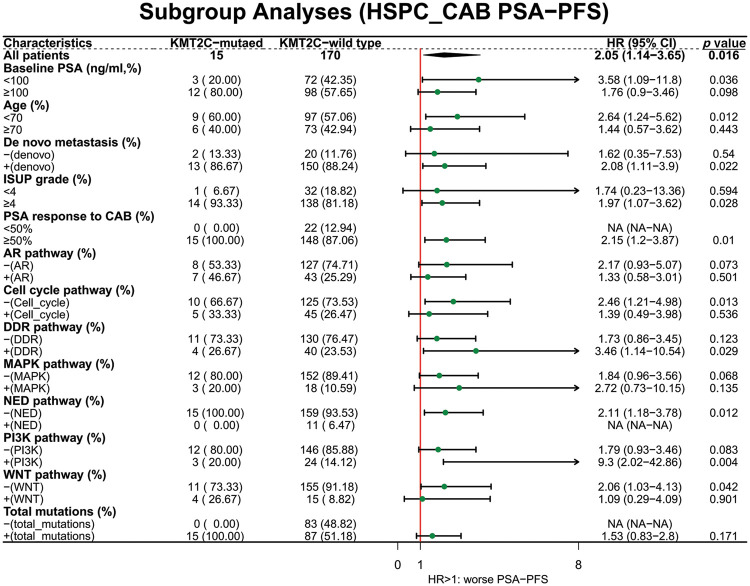
The prognostic value of *KMT2C* mutations in predicting the therapeutic efficacy of combined anti-androgen blockade in different clinicopathological subgroups. HSPC: hormone-sensitive prostate cancer; CAB: combined antiandrogen blockade; PSA: prostate-specific antigen; PSA-PFS: PSA progression-free survival; ISUP: International Society of Urological Pathology; AR: androgen receptor; DDR: DNA damage response; MAPK: mitogen-activated protein kinase; NED: neuroendocrine differentiation; PI3K: phosphatidylinositol 3-kinase; HR: hazard ratio; CI: confidence interval.

**SUPPLEMENTARY FIGURE 1:**
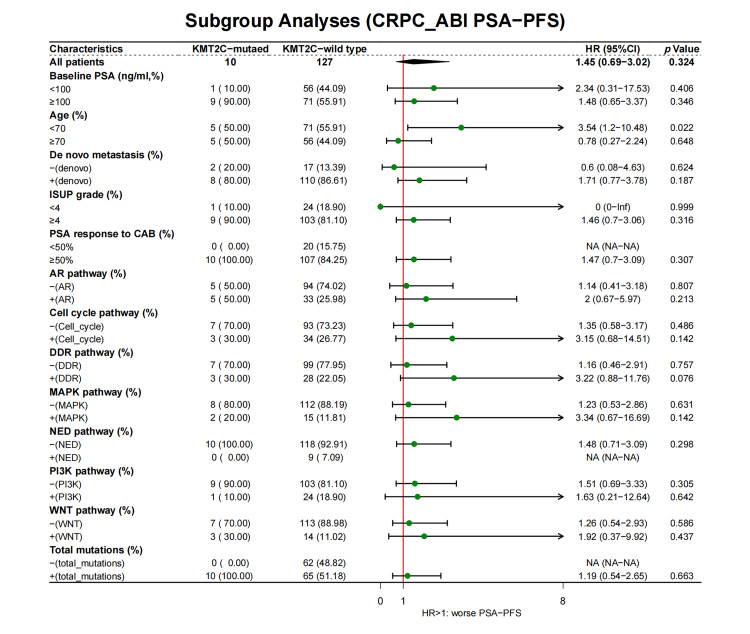
The prognostic value of KMT2C mutations in predicting the therapeutic efficacy of abiraterone in different clinicopathological subgroups.

We further evaluated the prognostic significance of *KMT2C* mutations in predicting the treatment efficacy at different disease stages. *KMT2C*-mutated patients had a significantly shorter PSA-PFS compared to *KMT2C*-WT patients when treated with CAB therapy during the HSPC phase (CAB PSA-PFS: mutated: 9.9 *vs*. WT: 17.6 months, *p* = 0.014, Fig. 3). *KMT2C* mutations were a significant prognosticator of PSA-PFS in the univariate analysis and had a borderline *p* value (*p* = 0.09) as well as the highest hazard ratio (HR = 1.84) in the multivariate analysis (Table 3). The predictive value of *KMT2C* mutations was also validated in patients of different subgroups. It could predict shorter PSA-PFS in 10 out of 23 subgroups while exhibiting a strong trend in the remaining subgroups (Fig. 4). In the ABI treatment, the CRPC patients with *KMT2C* mutations experienced a numerically shorter PSA-PFS (ABI PSA-PFS: mutated: 8.1 *vs*. WT 12.5 months, *p* = 0.32, Fig. 3). However, *KMT2C* mutations were not an independent factor in Cox regression analyses (Table 3). In subgroup analyses, the predictive significance of *KMT2C* mutations was only evident in the subgroup with age <70 (HR: 3.54, *p* = 0.022, Suppl. Fig. 1).

**Table 3:** Univariate and multivariate analyses of each factor’s value in predicting PSA-PFS of the CAB and abiraterone treatment

	HSPC_CAB PSA-PFS						CRPC_ABI PSA-PFS					
	Univariate			Multivariate			Univariate			Multivariate		
	HR	95.0% CI	*p*	HR	95.0% CI	*p*	HR	95.0% CI	*p*	HR	95.0% CI	*p*
KMT2C mutated	2.05	(1.14–3.65)	0.016	1.84	(0.91–3.71)	0.09	1.45	(0.69–3.02)	0.324	1.48	(0.62–3.54)	0.383
PSA ≥100 ng/mL	0.97	(0.69–1.35)	0.852	0.89	(0.62–1.27)	0.523	0.77	(0.52–1.16)	0.21	0.8	(0.5–1.29)	0.357
Age ≥70	0.89	(0.63–1.24)	0.476	0.79	(0.55–1.14)	0.207	1.16	(0.78–1.72)	0.467	1.2	(0.79–1.83)	0.396
*De novo* metastasis	1.19	(0.74–1.92)	0.471	1.21	(0.73–2)	0.47	0.84	(0.48–1.49)	0.557	0.76	(0.39–1.47)	0.408
ISUP ≥4	1.45	(0.94–2.26)	0.094	1.27	(0.81–2)	0.3	1.42	(0.82–2.46)	0.215	1.36	(0.75–2.47)	0.308
PSA response	0.81	(0.5–1.32)	0.4	0.95	(0.55–1.66)	0.87	0.85	(0.5–1.46)	0.56	0.77	(0.43–1.37)	0.373
AR pathway	1.88	(1.31–2.71)	0.001	1.41	(0.85–2.35)	0.181	1.15	(0.74–1.79)	0.532	0.64	(0.34–1.22)	0.173
Cell cycle pathway	1.26	(0.87–1.84)	0.225	0.78	(0.49–1.24)	0.3	1.68	(1.08–2.6)	0.021	1.28	(0.7–2.33)	0.429
DDR pathway	1.42	(0.98–2.08)	0.065	1.05	(0.65–1.69)	0.855	1.89	(1.2–2.98)	0.006	1.63	(0.93–2.86)	0.091
MAPK pathway	2.73	(1.64–4.55)	0	1.81	(0.95–3.45)	0.072	1.89	(1.07–3.37)	0.029	1.88	(0.88–4.04)	0.105
NED pathway	1.41	(0.71–2.77)	0.325	0.85	(0.38–1.9)	0.698	1.47	(0.68–3.19)	0.326	0.95	(0.32–2.85)	0.934
PI3K pathway	2.01	(1.29–3.11)	0.002	1.21	(0.64–2.29)	0.564	1.75	(1.07–2.86)	0.025	1.32	(0.66–2.64)	0.43
WNT pathway	2	(1.17–3.45)	0.012	1.31	(0.64–2.69)	0.46	1.2	(0.67–2.16)	0.534	0.74	(0.32–1.72)	0.489
Total mutations	1.63	(1.17–2.27)	0.004	1.14	(0.67–1.94)	0.625	1.63	(1.08–2.47)	0.02	1.29	(0.64–2.61)	0.476

## Discussion

This study investigates the prognostic significance of *KMT2C* mutations in a prostate cancer cohort. Our results suggested that *KMT2C* mutations were associated with *STK11* and *CTNNB1* mutations. *KMT2C*-mutated patients showed worse survival than *KMT2C*-WT patients in terms of both CRFS and OS, and *KMT2C* mutations were an independent risk factor in OS. Besides, *KMT2C* mutations were significantly associated with survival in certain patient subgroups, such as patients with PI3K and WNT pathway mutation, etc. Furthermore, *KMT2C* mutations were also associated with the treatment efficacy of prostate cancer patients.

KMT2C loss may exert a dual impact depending on the tumor context. Previous studies have shown that *KMT2C* mutations are linked to clinical outcomes and can be used for patient stratification for many tumors. In most cases, *KMT2C* mutations predict worse survival but can also indicate a better prognosis in certain cancer types [8–17]. Currently, there is no conclusion on the impact of *KMT2C* mutations in prostate cancer patients. Two *in vivo* studies showed conflicting results: one claimed KMT2C has a carcinogenic role and reported an overexpression in prostate cancer than in normal tissues (did not show survival data) [23]; another one claimed the truncated KMT2C a driver of tumor development and used public data to display a negative prognostic indication of KMT2C-loss [24]. Based on a relatively large population with balanced baseline characteristics, our results demonstrated that *KMT2C* mutations predicted poorer survival in prostate cancer patients and were an independent risk factor for OS. However, the discrepancies regarding the prognostic indication of *KMT2C* mutations in different cancer types and different experiment contexts require further exploration.

Our data also showed that *KMT2C* mutations were correlated with an unfavorable therapeutic response to androgen deprivation therapy and novel anti-androgen therapy. Interestingly, the association of *KMT2C* mutations with rapid progression and drug resistance in AR-directed treatment was only found in the HSPC stage, which prompts the use of liquid biopsy for early detection of *KMT2C* mutations in prostate cancer in order to provide in-time treatment outcome prediction. KMT2C is a known epigenetic regulator of gene expression. KMT2C regulates the DDR by its direct recruitment to DNA damage sites and *KMT2C* mutations are shown to disrupt homologous recombination (HR)–mediated DNA double-strand break repair, thus conferring PARPi sensitivity [18]. KMT2C also promotes the transcription of PD-L1 by binding to its enhancer and promoter in prostate cancer cells and regulating immune evasion through PD-L1 downregulation in mice [19]. Therefore, its loss-of-function possibly also sensitizes tumors to ICIs. Thus, we postulate that *KMT2C*-mutated cancer cells are likely to accumulate more DNA damage, possibly leading to high mutation loads and further promoting rapid progression and drug resistance. Therefore, with our knowledge of the complex impact of *KMT2C* mutations on therapeutic outcome, it is crucial to carefully consider treatment choices for this group of patients, thus stressing the significance of early detecting *KMT2C* mutations in prostate cancer patients.

We also noticed two gene mutations associated with *KMT2C* mutations: *STK11* and *CTNNB1*. *STK11* encodes a liver serine/threonine B1 kinase that is essential in many cellular processes and is involved in malignant metabolic transformations [32]. *STK11* is also considered a tumor suppressor in prostate cancer and is targeted by the diabetes drug metformin which has been shown to have anti-cancer activity [33,34]. *CTNNB1* encodes the pivotal protein β-catenin in the canonical WNT signaling, which is also frequently mutated in CRPC [35]. Further investigation showed that the association between *KMT2C* and *CTNNB1* existed only in patients who advanced to CRPC but not those who remained hormone-sensitive. This result indicates the necessity of looking into the synergistic effect and possible pharmaceutical combinations of *KMT2C*, *STK11*, and *CTNNB1* mutations. Also, the prognostic value of *KMT2C* mutations was more prominent in patients with PI3K and WNT pathway mutation. This finding reinforces the need for future studies to focus on *KMT2C*-mediated epigenetic regulation on PI3K and WNT pathways, both critical pathways in progressing to CRPC [32,35].

We used cfDNA-based liquid biopsy to detect the *KMT2C* mutations in this study. Several advantages of this method, including sensitivity, and the non-invasive and economical nature, have made it more prevalent than traditional tumor biopsies. Nowadays, in the context of personalized medicine, the need to weaponize liquid biopsies in favor of dynamic disease management is increasing. This study provides another illustration of using liquid biopsy results in clinical practice: *KMT2C* mutations could serve as a biomarker to predict unfavorable therapeutic efficacy and clinical outcomes; therefore, more aggressive treatment and closer follow-up will be needed for this group of patients.

## Supplementary Materials

SUPPLEMENTARY TABLE 1 Gene sequencing panel and list of genes included in each pathway.

SUPPLEMENTARY TABLE 2 Pathogenic (and likely pathogenic) variants detected in this study.

SUPPLEMENTARY TABLE 3 Baseline characteristics of patients who were still in HSPC stage and who had progressed to CRPC stage.

SUPPLEMENTARY TABLE 4 The association between KMT2C mutations and other gene mutations.

## Data Availability

The data that support the findings of this study are available from the corresponding author upon reasonable request.
