# Supplementary material for: Mutations in epigenetic regulator KMT2C detected by liquid biopsy are associated with worse survival in prostate cancer patients
Source: Oncol Res. 2023 Jun 27;31(4):605–14. doi: 10.32604/or.2023.028321 (PMC10319590; doi:10.32604/or.2023.028321)
Supplement: SUPPLEMENTARY TABLE 3 [file OncolRes-31-28321-s003.docx]

**Supplementary Table 3. Baseline characteristics of patients who were still in HSPC stage and who had progressed to CRPC stage.**

|  |  |  | **HSPC stage** | | | **CRPC stage** | | |
| --- | --- | --- | --- | --- | --- | --- | --- | --- |
|  |  | **Overall (n=221)** | **KMT2C-WT (n=54)** | **KMT2C-mutated (n=3)** | ***p* value** | **KMT2C-WT (n=151)** | **KMT2C-mutated (n=13)** | ***p* value** |
| **Baseline PSA (median [IQR])** | | 100.100 [48.000, 183.000] | 100.100 [43.098, 214.953] | 100.100 [92.050, 147.550] | 0.8857 | 100.100 [49.005, 163.550] | 100.100 [85.050, 100.100] | 0.9951 |
| **Baseline PSA (ng/mL, %)** | **<100** | 89 (40.27) | 17 (31.48) | 2 (66.67) | 0.5292 | 68 (45.03) | 2 (15.38) | 0.0748 |
|  | **≥100** | 132 (59.73) | 37 (68.52) | 1 (33.33) |  | 83 (54.97) | 11 (84.62) |  |
| **Age (median [IQR])** | | 69.000 [62.000, 75.000] | 69.500 [63.500, 74.750] | 62.000 [59.000, 71.000] | 0.6547 | 69.000 [62.000, 75.000] | 66.000 [59.000, 71.000] | 0.4464 |
| **Age (%)** | **<70** | 128 (57.92) | 33 (61.11) | 1 (33.33) | 0.7263 | 86 (56.95) | 8 (61.54) | 0.9773 |
|  | **≥70** | 93 (42.08) | 21 (38.89) | 2 (66.67) |  | 65 (43.05) | 5 (38.46) |  |
| **De novo metastasis (%)** | **No** | 24 (10.86) | 3 (5.56) | 0 (0.00) | 1 | 19 (12.58) | 2 (15.38) | 1 |
|  | **Yes** | 197 (89.14) | 51 (94.44) | 3 (100.00) |  | 132 (87.42) | 11 (84.62) |  |
| **PSA response to ADT (%)** | **<50%** | 28 (12.67) | 4 (7.41) | 0 (0.00) |  | 24 (15.89) | 0 (0.00) | 0.2514 |
|  | **≥50%** | 193 (87.33) | 50 (92.59) | 3 (100.00) | 1 | 127 (84.11) | 13 (100.00) | |
| **ISUP grade (%)** | **<4** | 41 (18.55) | 13 (24.07) | 0 (0.00) |  | 27 (17.88) | 1 (7.69) | 0.5805 |
|  | **≥4** | 180 (81.45) | 41 (75.93) | 3 (100.00) | 0.7945 | 124 (82.12) | 12 (92.31) |  |
| **AR pathway mutations (%)** | **No** | 167 (75.57) | 48 (88.89) | 3 (100.00) |  | 110 (72.85) | 6 (46.15) | 0.0869 |
|  | **Yes** | 54 (24.43) | 6 (11.11) | 0 (0.00) | 1 | 41 (27.15) | 7 (53.85) |  |
| **Cell cycle pathway mutations (%)** | **No** | 157 (71.04) | 40 (74.07) | 2 (66.67) |  | 107 (70.86) | 8 (61.54) | 0.6974 |
|  | **Yes** | 64 (28.96) | 14 (25.93) | 1 (33.33) | 1 | 44 (29.14) | 5 (38.46) |  |
| **DDR pathway mutations (%)** | **No** | 169 (76.47) | 43 (79.63) | 3 (100.00) |  | 114 (75.50) | 9 (69.23) | 0.8675 |
|  | **Yes** | 52 (23.53) | 11 (20.37) | 0 (0.00) | 0.9055 | 37 (24.50) | 4 (30.77) |  |
| **MAPK pathway mutations (%)** | **No** | 195 (88.24) | 49 (90.74) | 3 (100.00) | 1 | 133 (88.08) | 10 (76.92) | 0.4699 |
|  | **Yes** | 26 (11.76) | 5 (9.26) | 0 (0.00) |  | 18 (11.92) | 3 (23.08) |  |
| **NED pathway mutations (%)** | **No** | 209 (94.57) | 53 (98.15) | 3 (100.00) | 1 | 140 (92.72) | 13 (100.00) | 0.6674 |
|  | **Yes** | 12 (5.43) | 1 (1.85) | 0 (0.00) |  | 11 (7.28) | 0 (0.00) |  |
| **PI3K pathway mutations (%)** | **No** | 186 (84.16) | 53 (98.15) | 2 (66.67) | 0.2032 | 121 (80.13) | 10 (76.92) | 1 |
|  | **Yes** | 35 (15.84) | 2 (3.70) | 1 (33.33) |  | 30 (19.87) | 3 (23.08) |  |
| **WNT pathway mutations (%)** | **No** | 196 (88.69) | 52 (96.30) | 3 (100.00) | 1 | 132 (87.42) | 9 (69.23) | 0.1628 |
|  | **Yes** | 25 (11.31) | 21 (10.24) | 0 (0.00) |  | 19 (12.58) | 4 (30.77) |  |
| **Total mutations (%)** | **No** | 94 (42.53) | 27 (50.00) | 0 (0.00) | 0.2739 | 67 (44.37) | 0 (0.00) | 0.0047 |
|  | **Yes** | 127 (57.47) | 27 (50.00) | 3 (100.00) |  | 84 (55.63) | 13 (100.00) | |

HSPC: hormone-sensitive prostate cancer; CRPC: castration-resistant prostate cancer; PSA: prostate-specific antigen; ISUP: International Society of Urological Pathology; ADT: androgen deprivation therapy; AR: androgen receptor; DDR: DNA damage response; MAPK: mitogen-activated protein kinase; NED: neuroendocrine differentiation; PI3K: phosphatidylinositol 3-kinase; IQR: interquartile range; WT: wild type.
